# Abiraterone Acetate Versus Enzalutamide Against Chemo-Naïve Castration-Resistant Prostate Cancer With Full-Dose Induction

**DOI:** 10.7759/cureus.64217

**Published:** 2024-07-10

**Authors:** Tatsuya Shimomura, Keiichiro Mori, Akihiro Matsukawa, Wataru Fukuokaya, Takafumi Yanagisawa, Fumihiko Urabe, Masaya Murakami, Jun Miki, Hiroki Yamada, Takahiro Kimura

**Affiliations:** 1 Urology, Jikei University School of Medicine, Tokyo, JPN

**Keywords:** chemotherapy-naïve crpc, metastatic and non-metastatic crpc, survival outcome, psa response, arsi

## Abstract

Purpose

We recently released the multi-institutional real-world analysis about the difference in survival outcomes between abiraterone acetate and enzalutamide against chemo-naïve castration-resistant prostate cancer (CRPC) in a first-line setting. Although reduced dose induction cases were included in that analysis, induction dose reduction might correlate with reduced efficacy. In this study, we analyzed full-dose induction subgroups from our overall cohort and investigated the true difference in efficacy between these agents.

Methods

A total of 220 chemotherapy-naïve CRPC cases treated with full-dose induction of first-line androgen receptor signaling inhibitor (ARSI) were analyzed. Outcome measures were prostate-specific antigen (PSA) response, PSA progression-free survival (PSA-PFS), treatment failure-free survival (TFF), cancer-specific survival (CSS), and overall survival (OS).

Results

Abiraterone acetate and enzalutamide were administered to 58 and 162 patients, respectively. The median PSA response rate (−65.4% (A) and −81.5% (E), p = 0.0252), PSA decline ≥ 90% (22.4% (A) and 37.0% (E), p = 0.0478), PSA-PFS (median four months (A) and seven months (E), p = 0.00833), TFF (median six months (A) and 15 months (E), p<0.0001), CSS (median 45 months (A) and not reached (E), p < 0.0001), and OS (median 34 months (A) and 80 months (E), p<0.001) were significantly better in the E group.

Conclusion

This study showed that PSA response, PSA-PFS, TTF, CSS, and OS were better with first-line enzalutamide administration. Direct inhibition of androgen receptor signaling by enzalutamide is associated with better clinical outcomes in the full-dose induction cohort.

## Introduction

Prostate cancer is one of the most common cancers worldwide [1,2]. Usually, androgen deprivation therapy (ADT) is introduced at the start of treatment against advanced disease. However, cancer cells become resistant to ADT after one to four years, resulting in a progressive form of prostate cancer, called castration-resistant prostate cancer (CRPC) [3].

Abiraterone acetate and enzalutamide are categorized as new-generation androgen receptor signaling inhibitors (ARSI) although their therapeutic mechanisms are different [4-8]. Both agents were proven to the efficacy against metastatic CRPC both chemotherapy-naïve and post-chemotherapy CRPC by randomized controlled trials [7,9-11]. After significant survival benefits of introducing ARSI were shown in chemotherapy-naïve metastatic CRPC [10,11], these agents are introduced as a first-line setting against metastatic CRPC. Because of that, abiraterone acetate and enzalutamide have different hormonal mechanisms against prostate cancer, as mentioned above; which agent should be introduced first was one of the most important clinical problems. Therefore, we investigated our chemotherapy-naive CRPC cohort, and we released the multi-institutional real-world analysis about the difference in survival outcome between abiraterone acetate and enzalutamide as a first-line setting [12]. We concluded that enzalutamide induction correlated with significantly better survival outcomes in our multi-institutional retrospective study. Our real-world analysis included the cases with reduced induction dose in both abiraterone acetate and enzalutamide. However, it was reported that a reduced induction dose of ARSI correlated with reduced efficacy [13-15]. Therefore, we need to compare the full-dose induction cohort to analyze the true difference in the efficacy between abiraterone acetate and enzalutamide. In this study, we analyzed full-dose induction subgroups in our overall cohort and investigated the difference in the efficacy between abiraterone acetate and enzalutamide.

This article was previously posted at Research Square on April 18, 2024.

## Materials and methods

Patients and methods

A total of 242 chemotherapy-naïve CRPC patients introduced first-line abiraterone or enzalutamide between June 2014 and December 2016 at Jikei University Hospital and its affiliated institutions were included in our previous analysis, with a data cut-off date of November 2021 [12]. We reanalyzed full-dose induction subgroups from the overall cohort. The induction dose was 160 mg daily in enzalutamide or 1,000 mg daily in abiraterone acetate (plus 10 mg/daily of prednisolone). However, the dose was reduced, or the treatment agent was stopped temporarily, according to a patient’s medical condition during the clinical course if needed. Abiraterone acetate and enzalutamide are approved against both non-metastatic and metastatic CRPC in this country. Therefore, both non-metastatic and metastatic CRPC cases were included in this study. All patients with or without subsequent therapy were included in the study.

Parameters included in this analysis were initial prostate-specific antigen (iPSA), Gleason score (GS) at diagnosis of prostate cancer, age, Eastern Corporative Oncology Group performance status (PS), PSA at induction of ARSI, white blood cell (WBC) count, hemoglobin (Hb), alkaline phosphate (ALP), lactate dehydrogenase (LDH), C-reactive protein (CRP) (values < 0.04 or < 0.01 mg/dL were defined as 0.04 or 0.01 mg/dL, respectively), metastasis at the time of initiating androgen receptor-axis-targeted (ARAT) agents, primary ADT duration, time to the introduction of ARAT agents from the diagnosis of CRPC, and sequential treatment after failure of first-line ARAT agents. The outcome measures were PSA response rate, PSA decline ≥ 90%, PSA progression-free survival (PSA-PFS), treatment failure-free survival (TFF), cancer-specific survival (CSS), and overall survival (OS). PSA failure was defined according to the Prostate Cancer Working Group 2 (PCWC2) criteria. TFF was defined as the time to discontinue the agent due to clinical progression, the patient’s medical condition, or the physician’s judgment. TTF was the same as the treatment duration.

This study was approved by the Jikei University Institutional Review Board (34-189).

Statistical analysis

We performed the Mann-Whitney U test, t-test, or Fischer's exact test to evaluate the differences between the groups. Survival rates were estimated using the Kaplan-Meier method, and survival distributions were compared using the log-rank test. A Cox proportional hazard model was used for univariate and multivariate analyses. A multiple imputation method was used to complete the data. The inverse probability of treatment weighting (IPTW) approach was introduced to balance observable characteristics between the groups. Statistical significance was defined as a threshold p-value of <0.05. All statistical analyses were performed using R (The R Foundation for Statistical Computing, Vienna, Austria) and EZR (Saitama Medical Center, Jichi Medical University, Saitama, Japan) [16].

## Results

PSA response and survival outcome

Patient demographics of full-dose induction subgroups are summarized in Table 1. Abiraterone acetate and enzalutamide were introduced as first-line ARSI for 58 and 162 patients, respectively. The median follow-up duration was 24.0 months (range: 1-85 months).

**Table 1:** Patients' demographics *: at induction of ARSI, **: in the cohort of primary ARSI failure cases, iPSA: initial prostate-specific antigen, GS: Gleason score, ADT: androgen deprivation therapy, CRPC: castration-resistant prostate cancer, ARSI: androgen receptor signaling inhibitor, LN: lymph node, PSA: prostate-specific antigen, WBC: white blood cell, Hb: hemoglobin, ALP: alkaline phosphatase, LDH: lactate dehydrogenase, CRP: C-reactive protein

	ABI: 58 ENZ: 162
iPSA (ng/mL)	median 68.35 (1.07, 8736.00)
GS ≤ 7	41 (18.6%)
≥ 8	133 (60.5%)
NA	50 (21.8%)
Primary ADT (M)	median 19.00 (1, 221)
CRPC to ARSI (M)	median 2.00 (0, 103)
Age (years)	median 76.0 (49, 96)
Induction dose: full	220 (100%)
PS 0-1	213 (96.8%)
≥ 2	7 (3.2%)
LN metastasis* (-)	152 (69.1%)
(+)	52 (23.6%)
NA	16 (7.2%)
Metastasis* (-)	76 (34.5%)
(+)	129 (58.6%)
NA	15 (6.8%)
PSA (ng/mL)	median 12.78 (0.38, 8598.89)
WBC (μL)	median 6900 (3200, 19600)
Hb (g/dL)	median 12.50 (6.60, 16.00)
ALP (U/L)	median 249 (94, 3468)
LDH (U/L)	median 204 (113, 706)
CRP (ng/mL)	median 0.10 (0.01, 4.45)
2nd line treatment ** (-)	38 (23.0%)
(+)	125 (75.8%)
NA	2 (1.2%)
ARSI (2nd line)	93 (56.4%)
Docetaxel (2nd line)	25 (15.2%)
Other (2nd line)	7 (4.2%)

**Figure 1:**
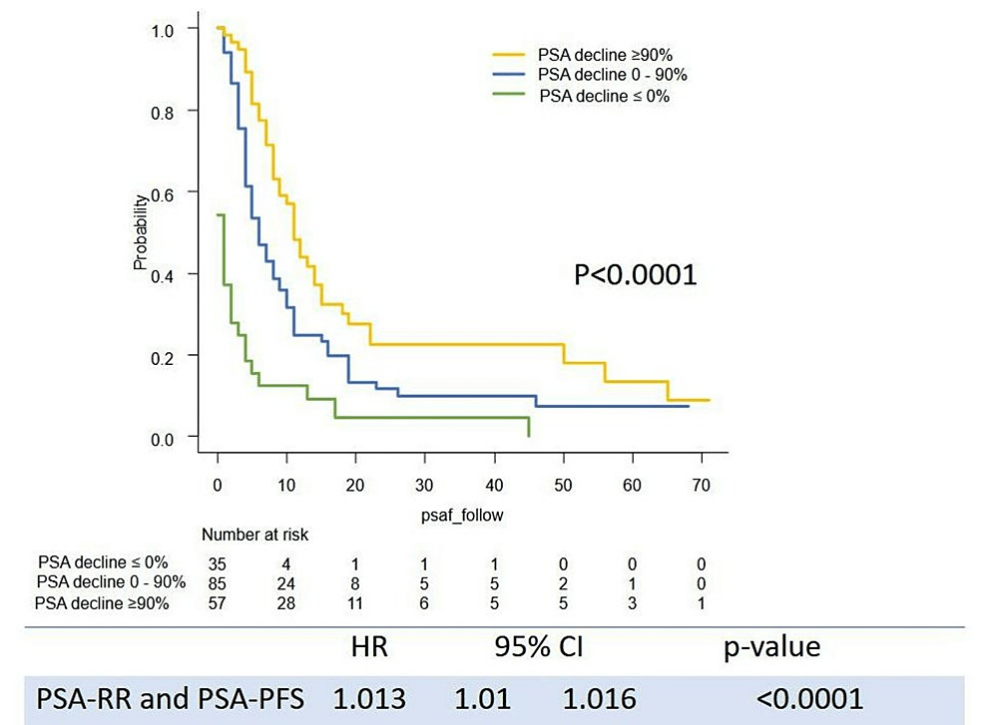
Kaplan–Meier curves of PSA-PFS stratified by the level of PSA decline PSA-PFS: PSA progression-free survival

**Figure 2:**
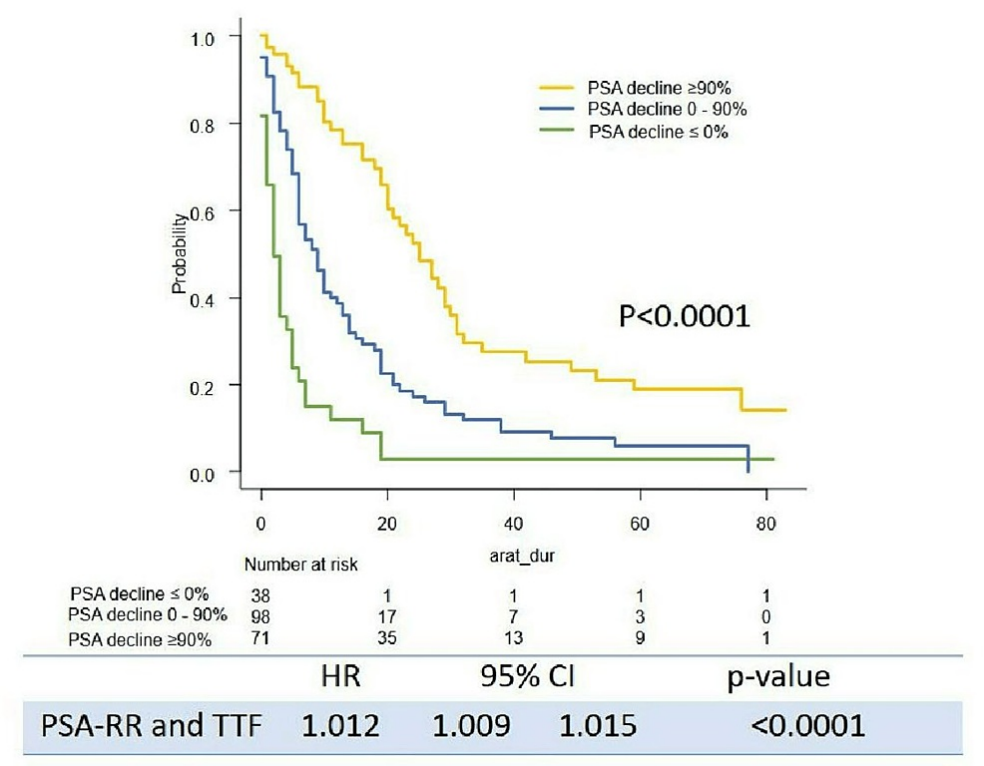
Kaplan–Meier curves of TFF stratified by the level of PSA decline TFF: treatment failure-free survival

**Figure 3:**
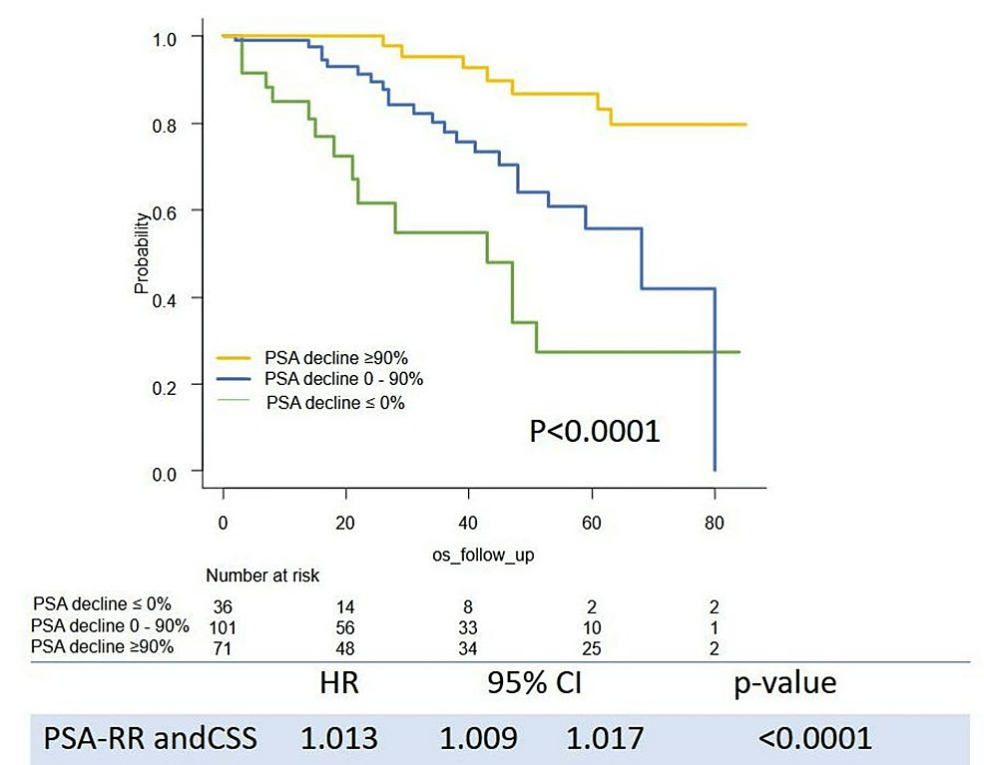
Kaplan–Meier curves of CSS stratified by the level of PSA decline CSS: cancer-specific survival

**Figure 4:**
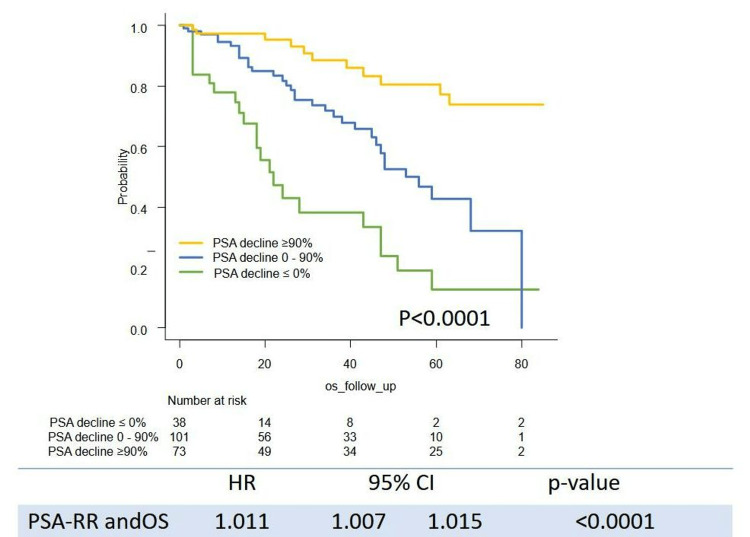
Kaplan–Meier curves of OS stratified by the level of PSA decline OS: overall survival

Survival outcome (PSA-PFS, TTF, CSS, and OS) stratified by the level of PSA decline are shown in Figures 1-4. Levels of PSA decline correlated survival outcome significantly (median PSA-PFS: PSA decline <0%: 1 m (95%CI: 0-2), 0-90%: 6 m (95%CI: 4-8), ≥90%: 11 m (95%CI: 8-15), p<0.0001; median TFF: PSA decline <0%: 2 m (95%CI: 1-4), 0-50%: 9 m (95%CI: 6-11), ≥90%: 25 m (95%CI: 20-30), p<0.0001; median CSS:PSA decline <0%:43 m (95%CI: 21-51), 0-90%: 68 m (95%CI: 48-NR), ≥90%: not reached (NR) (95%CI: NR-NR), p<0.0001; median OS: PSA decline <0%: 22 m (95%CI: 15-47), 0-50%: 53 m (95%CI: 45-NR), ≥90%: NR (95%CI: NR-NR), p<0.0001). The PSA response rate (PSA-RR) significantly correlated with PSA-PFS (p<0.0001), TFF (p<0.0001), CSS (p<0.0001), and OS (p<0.0001) (Figures 1-4). A better PSA response correlated with a better survival outcome.

Patient demographics of abiraterone acetate versus enzalutamide

Patient demographics (abiraterone acetate (A) versus enzalutamide (E)) are shown in Table 2. There was no significant difference in the baseline parameters at the time of diagnosis of prostate cancer and at the time of treatment initiation between the two groups, except for the visceral metastasis rate (19.0% (A) vs. 5.6% (E); p<0.05). Regarding sequential treatment, the second-line treatment induction rate was 82.2% (A) and 74.6% (E) (p=0.408) after primary ARSI failure. There were no significant differences in the induction rate of second-line ARSI and docetaxel between the groups. In terms of other second-line life-prolonging agents, second-line 223Ra was introduced one case in the abiraterone acetate group, and second-line 223Ra, bicalutamide, flutamide, estramustine phosphate, and ethinylestradiol were introduced two, one, one, one, and one case in the enzalutamide group, respectively.

**Table 2:** Patients' demographics abiraterone acetate vs enzalutamide *: at induction of ARSI, **: in the cohort of primary ARSI failure cases, iPSA: initial prostate-specific antigen, GS: Gleason score, ADT: androgen deprivation therapy, CRPC: castration-resistant prostate cancer, ARSI: androgen receptor signaling inhibitor, LN: lymph node, PSA: prostate-specific antigen, WBC: white blood cell, Hb: hemoglobin, ALP: alkaline phosphatase, LDH: lactate dehydrogenase, CRP: C-reactive protein

Factor	Abiraterone	Enzalutamide	p-value
n	58	162	
iPSA (ng/d)	Median 120.00 (1.07, 3434.27)	Median 52.10 (2.50, 8736.00)	0.097
GS ≤ 7	7 (12.1%)	34 (21.0%)	0.184
≥ 8	35 (60.3%)	98 (60.5%)	
NA	16 (27.6%)	30 (18.5%)	
Primary ADT(M)	Median 21.5 (3, 221)	Median 19.0 (1, 168)	0.53
CRPC to ARSI (M)	Median 5.0 (0, 54)	Median 2.0 (0, 103)	0.18
Age (years)	Median 77.0 (56, 89)	Median 75.0 (49, 96)	0.212
PS ≤ 1	56 (96.6%)	157 (96.9%)	1.00
≥ 2	2 (3.4%)	5 (3.1%)	
LN metastasis (-)	38 (65.5%)	114 (70.4%)	0.152
(+)	18 (31.0%)	34 (20.9%)	
NA	2 (3.4%)	14 (8.6%)	
Metastasis* (-)	19 (32.8%)	57 (35.9%)	0.523
(+)	38 (65.5%)	91 (55.8%)	
NA	1 (1.6%)	14 (8.3%)	
Bone metastasis * (-)	23 (39.7%)	62 (38.3%)	0.875
(+)	34 (58.6%)	86 (53.1%)	
NA	1 (1.7%)	14 (8.6%)	
Visceral metastasis * (-)	45 (77.6%)	139 (85.8%)	0.00897
(+)	11 (19.0%)	9 (5.6%)	
NA	2 (3.4%)	14 (8.6%)	
PSA (ng/mL)	Median 16.74 (1.43, 6504.38)	Median 11.38 (0.38, 8598.89)	0.077
WBC (/μL)	Median 6200 (3500, 19600)	Median 5900 (3200, 14000)	0.257
Hb (g/dL)	Median 12.60 (8.30, 16.00)	Median 12.50 (6.60, 15.60)	0.506
Plt (x10^4^ /μL)	Median 20.90 (10.2, 299.0)	Median 20.00 (8.90, 43.70)	0.056
ALP (U/L)	Median 248.5 (159, 1913)	Median 250.0 (94, 3468)	0.249
LDH (U/L)	Median 208 (146, 669)	Median 201 (113, 706)	0.059
CRP (mg/dL)	Median 0.16 (0.01, 4.45)	Median 0.10 (0.01, 3.99)	0.273
2nd line treatment (+) **	37 (82.2%)	88 (74.6%)	0.408
2nd line ARSI (+) **	26 (57.8%)	67 (56.8%)	0.862
2nd line docetaxel (+) **	10 (22.2%)	15 (12.7%)	0.145

PSA responses of first-line abiraterone acetate versus enzalutamide

**Figure 5:**
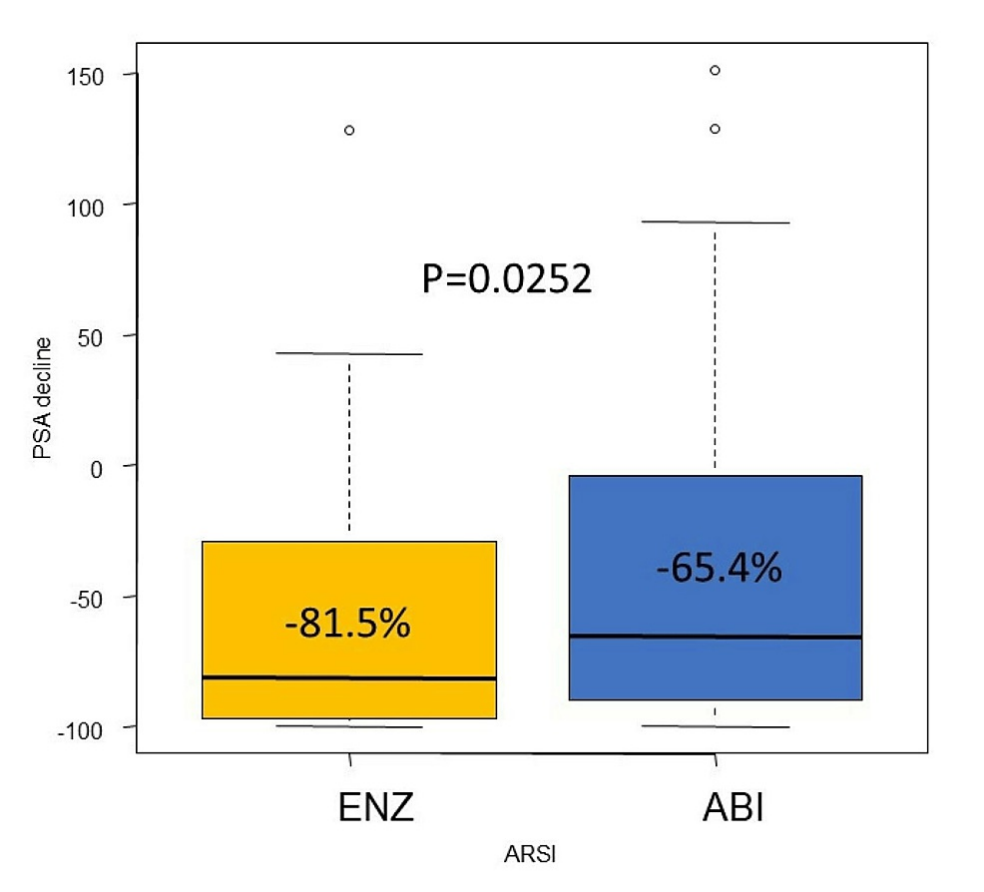
Median PSA decline of the abiraterone group and enzalutamide group ENZ: enzalutamide, ABI: abiraterone acetate

**Figure 6:**
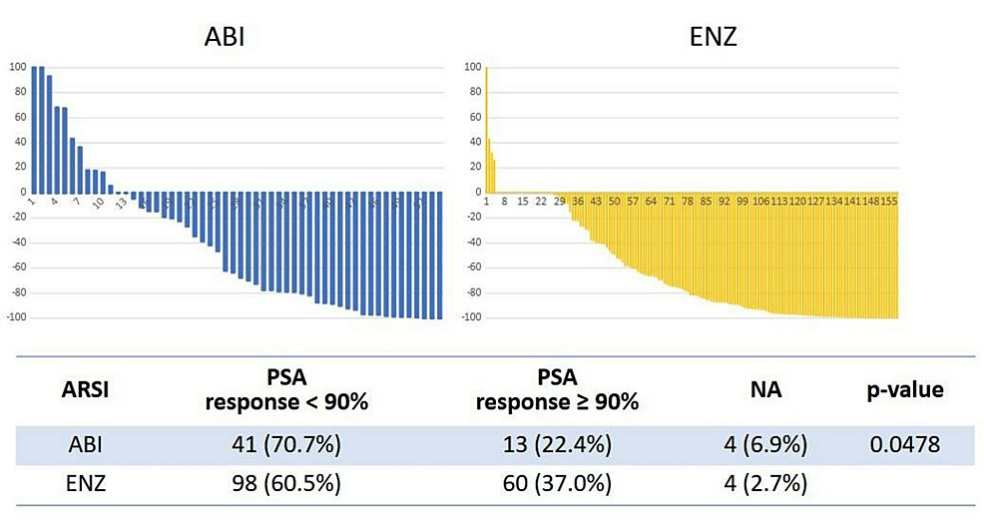
Waterfall plot of the best PSA response PSA: prostate-specific antigen, ARSI: androgen receptor signaling inhibitor, ABI: abiraterone acetate, ENZ: enzalutamide

In terms of PSA response rate, the median PSA response rates were −65.4% (A) and −81.5% (E) (p=0.0252) (Figure 5). A waterfall plot of the best PSA response in this cohort is shown in Figure 6. The PSA decline of ≥90% was 22.4% (A) and 37.0% (E) (p=0.0478). PSA responses were not available in four cases each, in both groups.

Survival outcome

**Figure 7:**
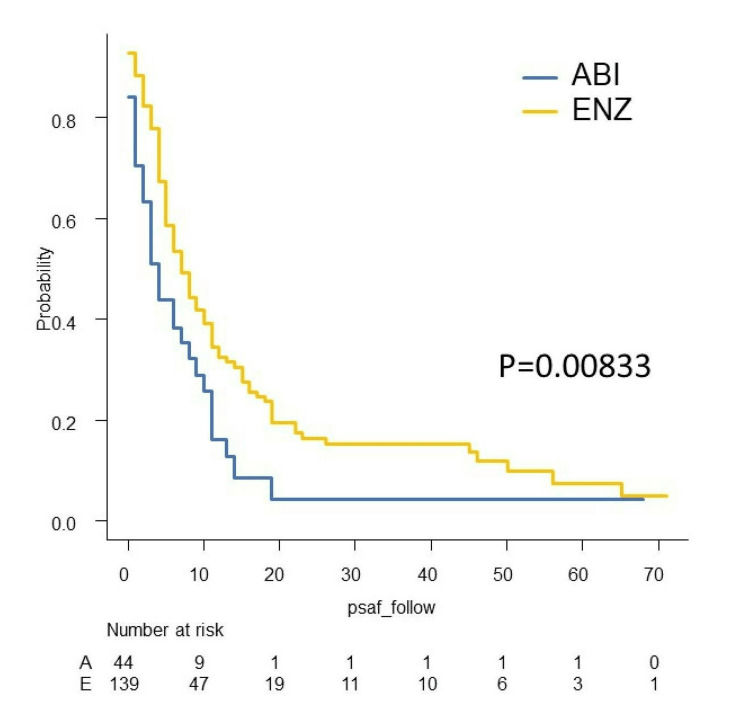
Kaplan–Meier curves of PSA-PFS in the abiraterone group and enzalutamide group PSA-PFS: PSA progression-free survival, ABI: abiraterone acetate, ENZ: enzalutamide

**Figure 8:**
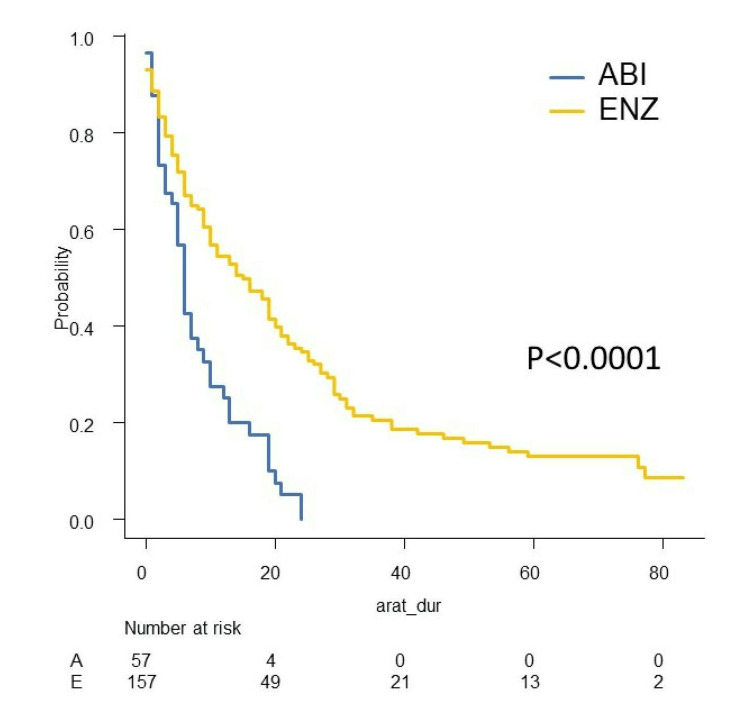
Kaplan–Meier curves of TFF in the abiraterone group and enzalutamide group TFF: treatment failure-free survival, ABI: abiraterone acetate, ENZ: enzalutamide

**Figure 9:**
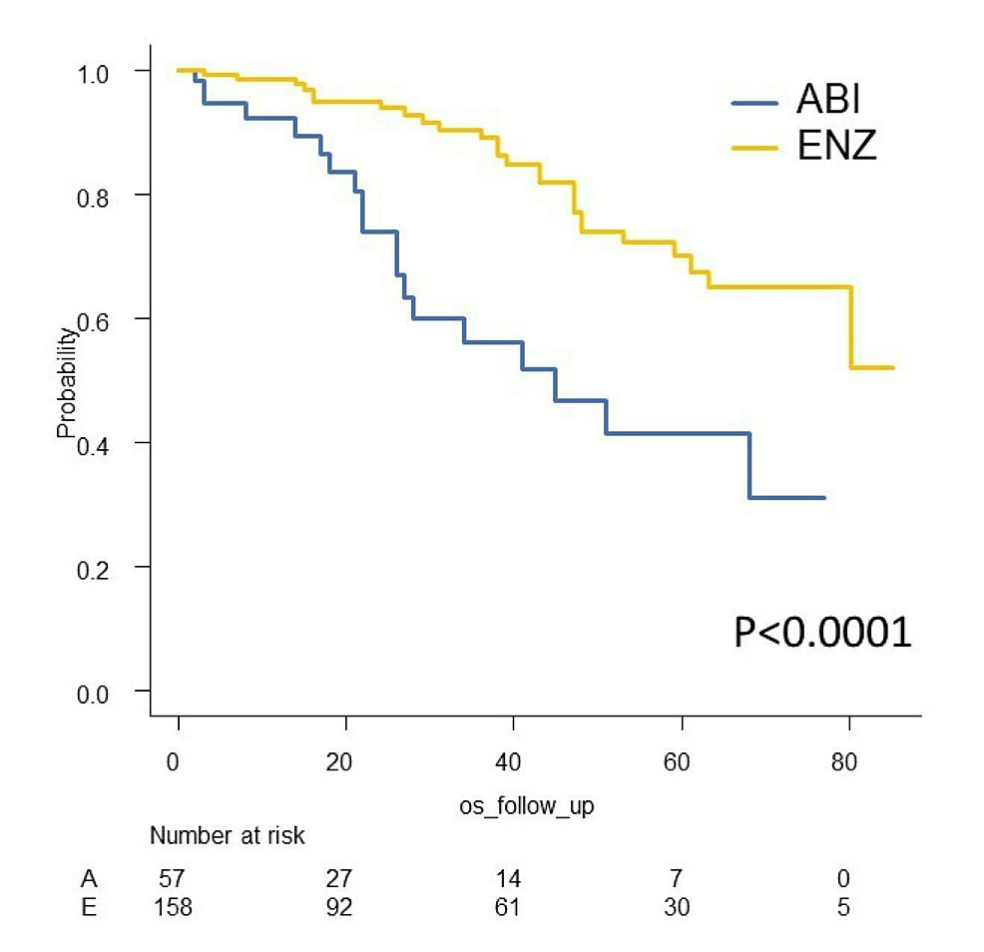
Kaplan–Meier curves of CSS in the abiraterone group and enzalutamide group CSS: cancer-specific survival, ABI: abiraterone acetate, ENZ: enzalutamide

**Figure 10:**
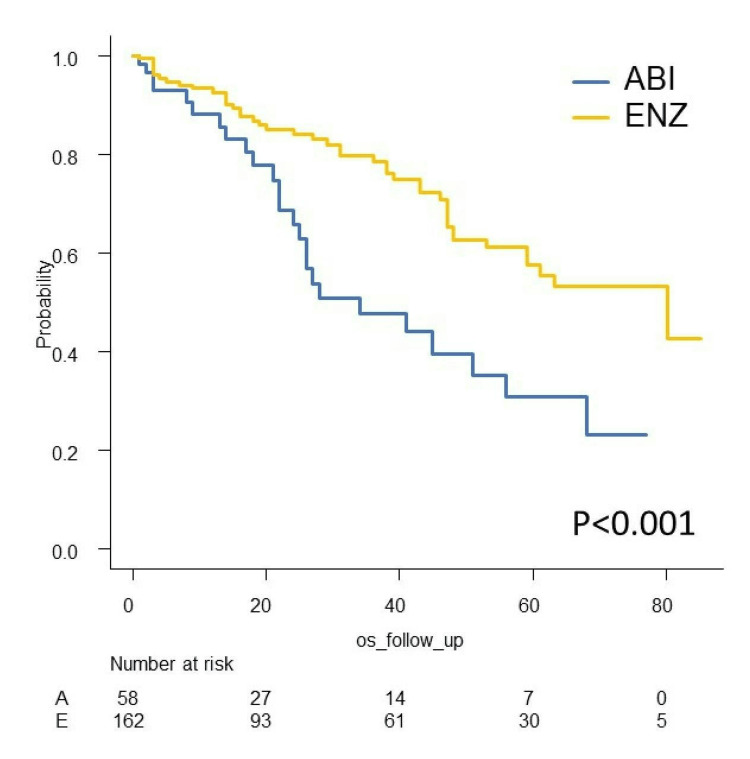
Kaplan–Meier curves of OS in the abiraterone group and enzalutamide group OS: overall survival, ABI: abiraterone acetate, ENZ: enzalutamide

We investigated PSA-PFS, TTF, CSS, and OS in this study. The Kaplan-Meier curves of PSA-PFS, TTF, CSS, and OS with respect to abiraterone acetate (A) versus enzalutamide (E) are shown in Figures 7-10. The median PSA-PFS (four months (A) and seven months (E); p=0.00833), median TFF (six months (A) and 15 months (E); p<0.0001), median CSS (45 months (A) and not reached (NR) (E); p<0.0001), and median OS (34 months (A) and 80 months (E); p<0.001) were better in the enzalutamide group.

Analyses of survival outcome using the IPTW method

**Figure 11:**
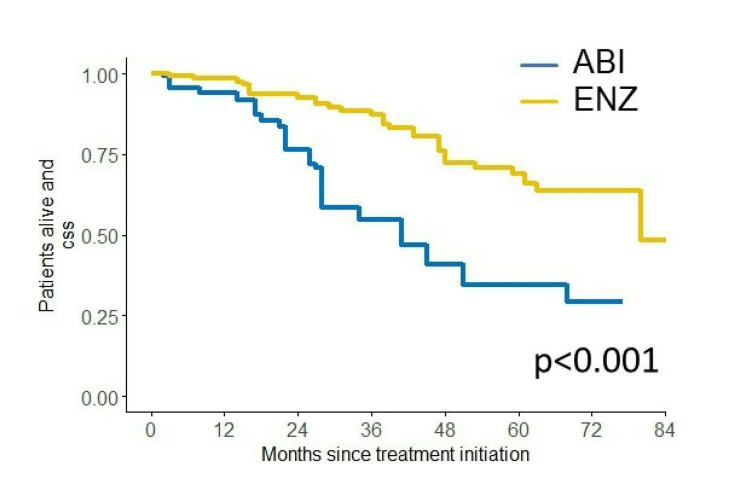
IPTW-adjusted Kaplan–Meier curves for CSS CSS: cancer-specific survival, ABI: abiraterone acetate, ENZ: enzalutamide

**Figure 12:**
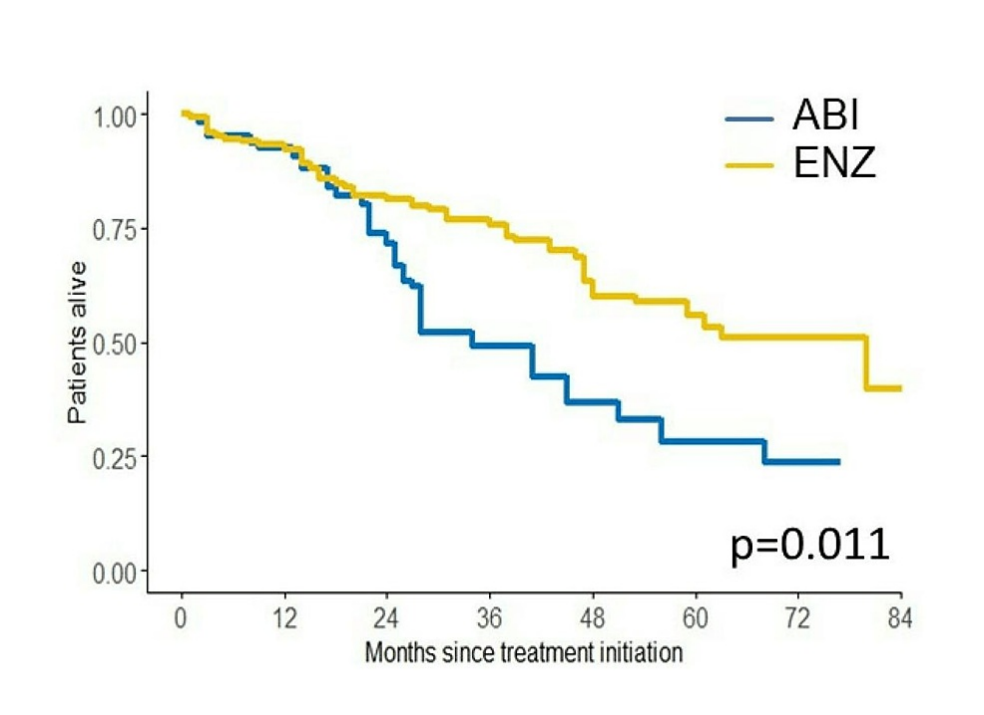
IPTW-adjusted Kaplan–Meier curves for OS OS: overall survival, ABI: abiraterone acetate, ENZ: enzalutamide

The IPTW method was used to minimize patients’ demographics. After IPTW adjustment, all absolute values of standardized mean difference (SMD) in weighted comparisons were <0.10, except for LDH (SMD=0.13(CSS) and -0.12 (OS)), which indicated that the distribution of baseline factors was similar between the abiraterone acetate and enzalutamide groups (Tables 3-4). The IPTW-adjusted Kaplan-Meier curves show that the median CSS and OS were significantly longer in the enzalutamide group (41 months (A) and 80 months (E), p<0.001 (CSS); 34 months (A) and 80 months (E), p=0.011 (OS)) (Figures 11-12).

**Table 3:** Before and after the IPTW-adjusted baseline demographics for CSS ^1^: mean (SD); n (%) ^2^: Standardized mean difference ^3^: mean (SD); % *: log (value), iPSA: initial prostate-specific antigen, GS: Gleason score, ADT: androgen deprivation therapy, CRPC: castration-resistant prostate cancer, ARSI: androgen receptor signaling inhibitor, PSA: prostate-specific antigen, WBC: white blood cell, Hb: hemoglobin, ALP: alkaline phosphatase, LDH: lactate dehydrogenase, CRP: C-reactive protein

Characteristic	Unweighted population (before imputation)				Unweighted population (after imputation)				Weighted population		
	Overall, N = 215^1^	ABI, N = 57^1^	ENZ, N = 158^1^	Difference^2^	Overall, N = 10,750^1^	ABI, N = 2,850^1^	ENZ, N = 7,900^1^	Difference^2^	ABI, N = 2,810^3^	ENZ, N = 7,937^3^	Difference^2^
iPSA	489.6 (1,200.4)	397.0 (710.5)	523.5 (1,336.0)	-0.12	490.4 (1,197.2)	392.0 (699.5)	525.8 (1,330.1)	-0.13	463.0 (826.8)	486.4 (1,299.4)	-0.02
NA	6 (2.8)	1 (1.8)	5 (3.2)								
PSA	108.4 (737.2)	171.7 (873.1)	86.0 (684.4)	0.11	108.2 (733.9)	169.9 (858.1)	86.0 (682.2)	0.11	102.5 (602.1)	101.4 (769.6)	0
NA	1 (0.5)	1 (1.8)	0 (0)								
ECOG performance status				0.06	75.4 (7.7)	76.2 (7.1)	75.1 (7.8)	0.06	75.3 (7.2)	75.4 (7.7)	-0.06
≤ 1	209 (97.2)	55 (96.5)	154 (97.5)		10,450 (97.2)	2,750 (96.5)	7,700 (97.5)		98.1	97.3	
≥ 2	6 (2.8)	2 (3.5)	4 (2.5)		300 (2.8)	100 (3.5)	200 (2.5)	0.06	1.9	2.7	
Age at treatment initiation	75.4 (7.7)	76.2 (7.2)	75.1 (7.8)	0.15	75.4 (7.7)	76.2 (7.1)	75.1 (7.8)	0.15	75.3 (7.2)	75.4 (7.7)	-0.01
NA	1 (0.5)	0 (0)	1 (0.6)						1.9	2.7	
Gleason score				0.24				0.2			0.01
≤ 7	41 (24.1)	7 (16.7)	34 (26.6)		2,630 (24.5)	522 (18.3)	2,108 (26.7)		25.1	25.7	
≥ 8	129 (75.9)	35 (83.3)	94 (73.4)		8,120 (75.5)	2,328 (81.7)	5,792 (73.3)		74.9	74.3	
NA	45 (21)	15 (26)	30 (19)								
Bone metastasis				0.04				0.02			0.05
Negative	85 (42.5)	23 (41.1)	62 (43.1)		4,486 (41.7)	1,164 (40.8)	3,322 (42.1)		38.7	41.1	
Positive	115 (57.5)	33 (58.9)	82 (56.9)		6,264 (58.3)	1,686 (59.2)	4,578 (57.9)		61.3	58.9	
NA	15 (7.0)	1 (1.8)	14 (8.9)								
Lymph node metastasis				0.24				0.23			0.05
Negative	149 (74.9)	37 (67.3)	112 (77.8)		8,026 (74.7)	1,918 (67.3)	6,108 (77.3)		72.6	74.6	
Positive	50 (25.1)	18 (32.7)	32 (22.2)		2,724 (25.3)	932 (32.7)	1,792 (22.7)		27.4	25.4	
NA	16 (7.4)	2 (3.5)	14 (8.9)								
Visceral metastasis				0.47				0.48			0.03
Negative	181 (91.0)	44 (80.0)	137 (95.1)		9,787 (91.0)	2,273 (79.8)	7,514 (95.1)		89.5	90.3	
Positive	18 (9.0)	11 (20.0)	7 (4.9)		963 (9.0)	577 (20.2)	386 (4.9)		10.5	9.7	
NA	16 (7.4)	2 (3.5)	14 (8.9)								
Any metastasis				0.12				0.1			0.06
Negative	76 (38.0)	19 (33.9)	57 (39.6)		3,994 (37.2)	962 (33.8)	3,032 (38.4)		33.6	36.5	
Positive	124 (62.0)	37 (66.1)	87 (60.4)		6,756 (62.8)	1,888 (66.2)	4,868 (61.6)		66.4	63.5	
NA	15 (7.0)	1 (1.8)	14 (8.9)								
Duration of ADT	29.9 (32.3)	29.3 (31.1)	30.2 (32.9)	-0.03	30.3 (32.8)	31.1 (33.1)	30.0 (32.7)	0.03	28.3 (31.9)	29.7 (32.1)	-0.04
NA	22 (10)	6 (11)	16 (10)								
Baseline hemoglobin	12.3 (1.5)	12.4 (1.7)	12.3 (1.5)	0.09	12.3 (1.5)	12.4 (1.6)	12.3 (1.4)	0.05	12.3 (1.6)	12.3 (1.5)	0
NA	20 (9.3)	11 (19)	9 (5.7)								
Baseline alkaline phosphatase*	5.6 (0.6)	5.7 (0.5)	5.6 (0.6)	0.11	5.6 (0.6)	5.7 (0.5)	5.6 (0.6)	0.14	5.7 (0.5)	5.6 (0.6)	0.02
NA	27 (13)	12 (21)	15 (9.5)								
Baseline lactate dehydrogenase*	5.3 (0.3)	5.4 (0.3)	5.3 (0.2)	0.35	5.3 (0.3)	5.4 (0.3)	5.3 (0.2)	0.35	5.4 (0.2)	5.3 (0.2)	0.13
NA	20 (9.3)	10 (18)	10 (6.3)								
Baseline C-reactive protein*	-1.9 (1.3)	-1.7 (1.3)	-1.9 (1.3)	0.21	-1.9 (1.3)	-1.6 (1.3)	-1.9 (1.3)	0.21	-1.9 (1.4)	-1.9 (1.3)	-0.03
NA	58 (27)	20 (35)	38 (24)								

**Table 4:** Before and after the IPTW-adjusted baseline demographics for OS ^1^: mean (SD); n (%) ^2^: standardized mean difference ^3^: mean (SD); % *: log (value), iPSA: initial prostate-specific antigen, GS: Gleason score, ADT: androgen deprivation therapy, CRPC: castration-resistant prostate cancer, ARSI: androgen receptor signaling inhibitor, PSA: prostate-specific antigen, WBC: white blood cell, Hb: hemoglobin, ALP: alkaline phosphatase, LDH: lactate dehydrogenase, CRP: C-reactive protein

Characteristic	Unweighted population (before imputation)				Unweighted population (after imputation)				Weighted population		
	Overall, N = 220^1^	ABI, N = 58^1^	ENZ, N = 162^1^	Difference^2^	Overall, N = 11,000^1^	ABI, N = 2,900^1^	ENZ, N = 8,100^1^	Difference^2^	ABI, N = 2,844^3^	ENZ, N = 8,139^3^	Difference^2^
iPSA	489.2 (1,192.0)	394.0 (704.5)	523.8 (1,325.7)	-0.12	491.0 (1,188.7)	389.2 (694.3)	527.4 (1,319.6)	-0.13	472.8 (829.1)	491.1 (1,299.6)	-0.02
NA	6 (2.7)	1 (1.7)	5 (3.1)								
PSA	108.8 (730.7)	171.7 (873.1)	87.1 (676.4)	0.11	109.6 (731.0)	172.2 (867.0)	87.1 (674.3)	0.11	106.9 (627.0)	104.7 (777.0)	0
NA	2 (0.9)	2 (3.4)	0 (0)								
ECOG performance status				0.02				0.02			-0.05
≤ 1	213 (96.8)	56 (96.6)	157 (96.9)		10,650 (96.8)	2,800 (96.6)	7,850 (96.9)		97.4	96.6	
≥ 2	7 (3.2)	2 (3.4)	5 (3.1)		350 (3.2)	100 (3.4)	250 (3.1)		2.6	3.4	
Age at treatment initiation	75.4 (7.7)	76.4 (7.2)	75.0 (7.8)	0.18	75.4 (7.7)	76.4 (7.2)	75.0 (7.8)	0.18	75.1 (7.3)	75.3 (7.8)	-0.02
NA	1 (0.5)	0 (0)	1 (0.6)								
Gleason score				0.22				0.17			0.02
≤ 7	41 (23.6)	7 (16.7)	34 (25.8)		2,652 (24.1)	551 (19.0)	2,101 (25.9)		23.9	24.9	
≥ 8	133 (76.4)	35 (83.3)	98 (74.2)		8,348 (75.9)	2,349 (81.0)	5,999 (74.1)		76.1	75.1	
NA	46 (21)	16 (28)	30 (19)								
Bone metastasis				0.03				0.02			0.03
Negative	85 (41.5)	23 (40.4)	62 (41.9)		4,486 (40.8)	1,166 (40.2)	3,320 (41.0)		38.8	40.3	
Positive	120 (58.5)	34 (59.6)	86 (58.1)		6,514 (59.2)	1,734 (59.8)	4,780 (59.0)		61.2	59.7	
NA	15 (6.8)	1 (1.7)	14 (8.6)								
Lymph node metastasis				0.21				0.19			0.05
Negative	152 (74.5)	38 (67.9)	114 (77.0)		8,151 (74.1)	1,963 (67.7)	6,188 (76.4)		71.1	73.5	
Positive	52 (25.5)	18 (32.1)	34 (23.0)		2,849 (25.9)	937 (32.3)	1,912 (23.6)		28.9	26.5	
NA	16 (7.3)	2 (3.4)	14 (8.6)								
Visceral metastasis				0.41				0.43			0.04
Negative	184 (90.2)	45 (80.4)	139 (93.9)		9,927 (90.2)	2,314 (79.8)	7,613 (94.0)		88	89.3	
Positive	20 (9.8)	11 (19.6)	9 (6.1)		1,073 (9.8)	586 (20.2)	487 (6.0)		12	10.7	
NA	16 (7.3)	2 (3.4)	14 (8.6)								
Any metastasis				0.11				0.09			0.04
Negative	76 (37.1)	19 (33.3)	57 (38.5)		3,999 (36.4)	960 (33.1)	3,039 (37.5)		34	35.8	
Positive	129 (62.9)	38 (66.7)	91 (61.5)		7,001 (63.6)	1,940 (66.9)	5,061 (62.5)		66	64.2	
NA	15 (6.8)	1 (1.7)	14 (8.6)								
Duration of ADT	30.6 (34.7)	32.9 (40.7)	29.8 (32.5)	0.09	30.9 (35.3)	34.4 (41.8)	29.7 (32.6)	0.13	28.6 (35.7)	29.9 (32.6)	-0.04
NA	22 (10)	6 (10)	16 (9.9)								
Baseline hemoglobin	12.3 (1.6)	12.4 (1.8)	12.2 (1.5)	0.07	12.3 (1.6)	12.3 (1.7)	12.3 (1.5)	0.02	12.3 (1.6)	12.3 (1.6)	0
NA	20 (9.1)	11 (19)	9 (5.6)								
Baseline alkaline phosphatase*	5.6 (0.6)	5.7 (0.5)	5.6 (0.6)	0.14	5.6 (0.6)	5.7 (0.5)	5.6 (0.6)	0.17	5.7 (0.5)	5.6 (0.6)	0.04
NA	27 (12)	12 (21)	15 (9.3)								
Baseline lactate dehydrogenase*	5.3 (0.3)	5.4 (0.3)	5.3 (0.3)	0.34	5.3 (0.3)	5.4 (0.3)	5.3 (0.3)	0.36	5.4 (0.2)	5.3 (0.3)	0.12
NA	20 (9.1)	10 (17)	10 (6.2)								
Baseline C-reactive protein*	-1.8 (1.3)	-1.6 (1.3)	-1.9 (1.3)	0.18	-1.8 (1.3)	-1.6 (1.3)	-1.9 (1.3)	0.2	-1.8 (1.4)	-1.8 (1.4)	-0.02
NA	58 (26)	20 (34)	38 (23)								

Univariate and multivariate analyses for OS and CSS

Univariate and multivariate analyses for CSS and OS are shown in Tables 5-6. In univariate analysis for CSS, age, PSA, Hb, ALP, and ARSI (p=0.0461, <0.0001, 0.017, <0.001, and <0.001, respectively) were significant. In the multivariate analysis for CSS, ALP (p<0.01) and ARSI (enzalutamide vs. abiraterone acetate, p<0.01) were significant factors (Table 5). In univariate analysis for OS, age, PS, metastasis, PSA, Hb, ALP, LDH, and ARSI (p=0.012, 0.0024, 0.023, <0.001, <0.0001, 0.0045, <0.001, and 0.00126, respectively) were significant factors. In the multivariate analysis for OS, Hb (p<0.01) and ARSI (enzalutamide vs. abiraterone acetate, p=0.0155) were significant factors (Table 6).

**Table 5:** Univariate and multivariate analyses for CSS iPSA: initial prostate-specific antigen, GS: Gleason score, ADT: androgen deprivation therapy, CRPC: castration-resistant prostate cancer, ARSI: androgen receptor signaling inhibitor, LN: lymph node, PSA: prostate-specific antigen, WBC: white blood cell, Hb: hemoglobin, ALP: alkaline phosphatase, LDH: lactate dehydrogenase, CRP: C-reactive protein

	Univariate	Multivariate			
	p-value	HR	95% CI		p-value
iPSA	0.6793				
GS	0.4025				
Primary ADT	0.8586				
CRPC to ARSI	0.3670				
Age	0.0461	1.043	0.9965	1.091	0.071
PS	0.1719				
LN metastasis	0.08497				
Metastasis	0.1014				
(Bone metastasis)	0.1837				
(Visceral metastasis)	0.9867				
PSA	<0.0001	1.000	0.9999	1.000	0.0972
WBC	0.7994				
Hb	0.01705	0.8358	0.6671	1.0470	0.119
PLT	0.8360				
ALP	<0.001	1.001	1.000	1.001	0.003445
LDH	0.07907				
CRP	0.7340				
ARSI	<0.001	0.369	0.1735	0.6545	0.001321

**Table 6:** Univariate and multivariate analyses for OS iPSA: initial prostate-specific antigen, GS: Gleason score, ADT: androgen deprivation therapy, CRPC: castration-resistant prostate cancer, ARSI: androgen receptor signaling inhibitor, LN: lymph node, PSA: prostate-specific antigen, WBC: white blood cell, Hb: hemoglobin, ALP: alkaline phosphatase, LDH: lactate dehydrogenase, CRP: C-reactive protein

	Univariate	Multivariate			
	p-value	HR	95% CI		p-value
iPSA	0.651				
GS	0.09014				
Primary ADT	0.8929				
CRPC to ARSI	0.2734				
Age	0.01185	1.0260	0.9991	1.065	0.1673
PS	0.02353	1.280	0.4075	3.700	0.7154
LN metastasis	0.05604				
Metastasis	0.02275	1.6080	0.9045	2.860	0.1056
(Bone metastasis)	(0.08547)				
(Visceral metastasis)	(0.3365)				
PSA	<0.001	1.000	0.9998	1.000	0.4153
WBC	0.2876				
Hb	<0.0001	0.7779	0.6489	0.9325	0.006609
PLT	0.9932				
ALP	0.0045	1.000	0.9997	1.001	0.2813
LDH	<0.001	1.003	1.000	1.005	0.05413
CRP	0.849				
ARSI	0.001257	0.49970	0.2821	0.8755	0.01552

Second-line treatment and survival outcome

**Figure 13:**
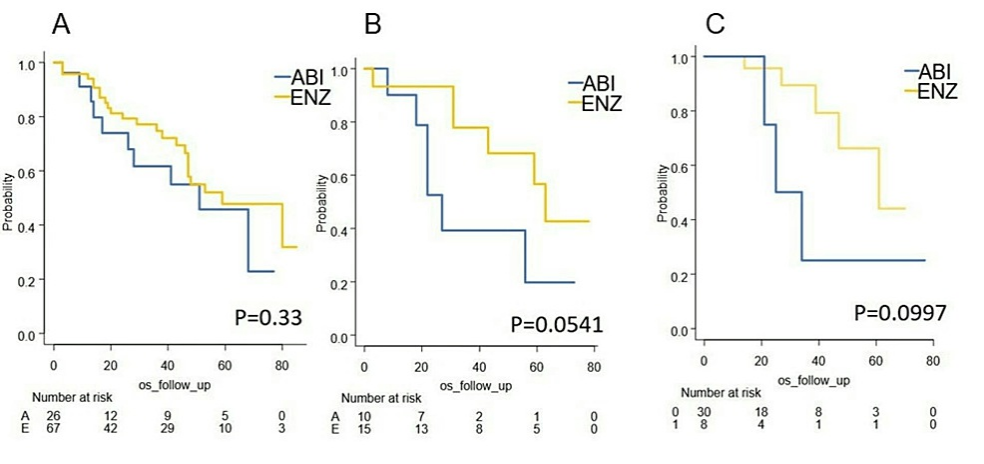
Second-line treatment and survival outcome A: Kaplan–Meier curves of OS in the 2nd-line ARSI cohort, B: Kaplan–Meier curves of OS in the 2nd-line docetaxel cohort, C: Kaplan–Meier curves of OS in the cohort without 2nd-line treatment after primary ABI: abiraterone acetate, ENZ: enzalutamide

Correlations between second-line treatment and survival outcome were analyzed. In the ARSI-ARSI sequent cohort, the OS was not different between the groups (median OS was 51 months (A) and 59 months (E) (p=0.33) (Figure 13A). In the ARSI-docetaxel cohort, although it did not reach a significant difference (p=0.0541), OS trended better in the enzalutamide group (median OS was 27 months (A) and 63 months (E) (p=0.0541) (Figure 13B). In the cohort without subsequent therapy after failure of primary ARSI, although it did not reach a significant difference (p=0.0997), the OS of the enzalutamide group might be better (median OS was 29.5 months (A) and 61 months (E) (Figure 13C).

Survival outcome of nmCRPC and mCRPC

**Figure 14:**
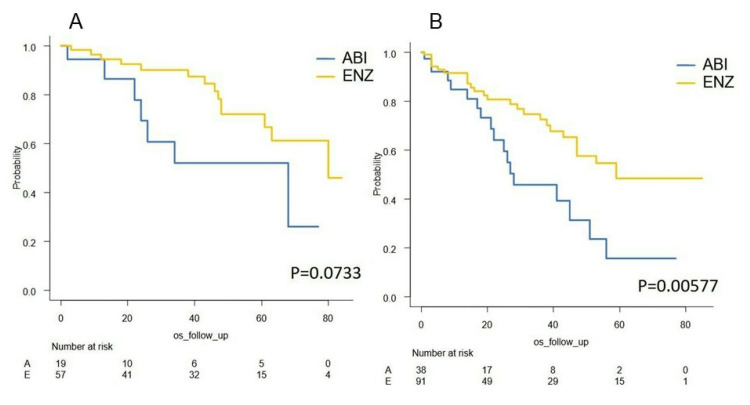
Kaplan–Meier curve of the OS in metastatic and non-metastatic CRPC A: Kaplan–Meier curve of the OS in the non-metastatic CRPC subgroup B: Kaplan–Meier curve of the OS in the metastatic CRPC subgroup ABI: abiraterone acetate, ENZ: enzalutamide

The OS of non-metastatic CRPC (nmCRPC) and metastatic CRPC (mCRPC) are shown in Figure 14. The median OS of non-metastatic disease was 68 months (abiraterone) and 80 months (enzalutamide) (p=0.0733, Figure 14A), and the median OS of metastatic disease was 28 months (abiraterone) and 59 months (enzalutamide) (p=0.00577, Figure 14B).

Subgroup analysis

**Figure 15:**
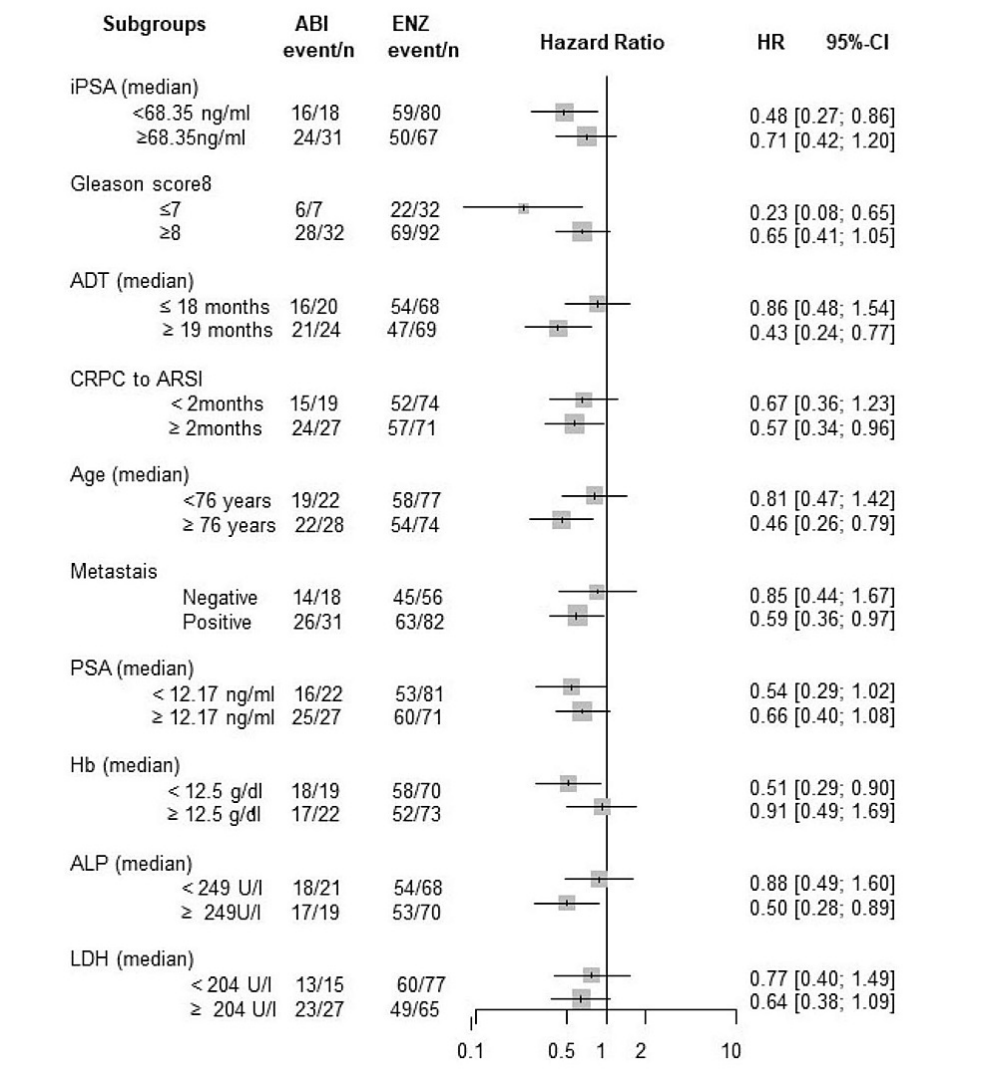
Forrest plot of the subgroup analysis for PSA-PFS PSA-PFS: PSA progression-free survival, iPSA: initial prostate-specific antigen, GS: Gleason score, ADT: androgen deprivation therapy, CRPC: castration-resistant prostate cancer, ARSI: androgen receptor signaling inhibitor, LN: lymph node, PSA: prostate-specific antigen, WBC: white blood cell, Hb: hemoglobin, ALP: alkaline phosphatase, LDH: lactate dehydrogenase, CRP: C-reactive protein

**Figure 16:**
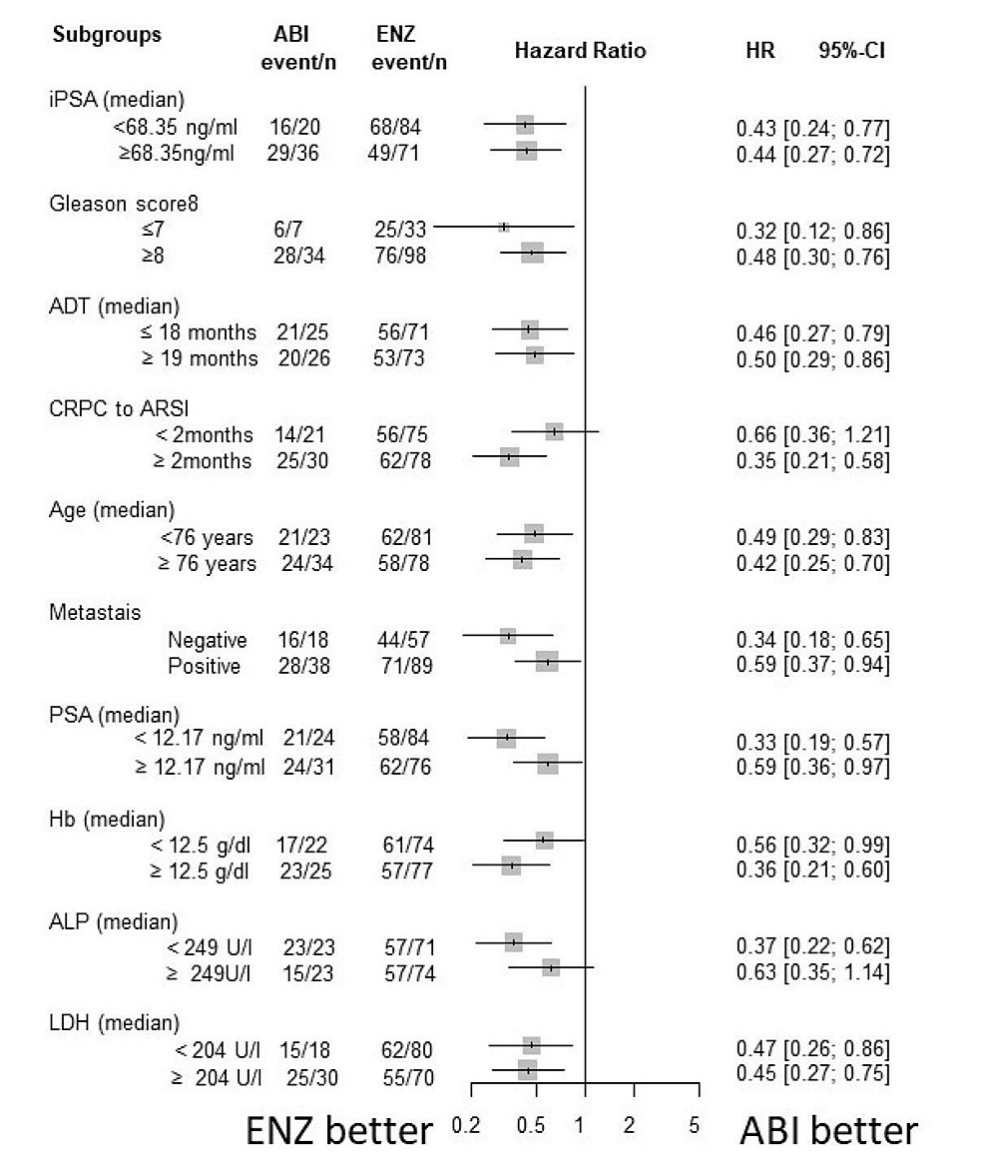
Forrest plot of the subgroup analysis for TFF TFF: treatment failure-free survival, iPSA: initial prostate-specific antigen, GS: Gleason score, ADT: androgen deprivation therapy, CRPC: castration-resistant prostate cancer, ARSI: androgen receptor signaling inhibitor, LN: lymph node, PSA: prostate-specific antigen, WBC: white blood cell, Hb: hemoglobin, ALP: alkaline phosphatase, LDH: lactate dehydrogenase, CRP: C-reactive protein

**Figure 17:**
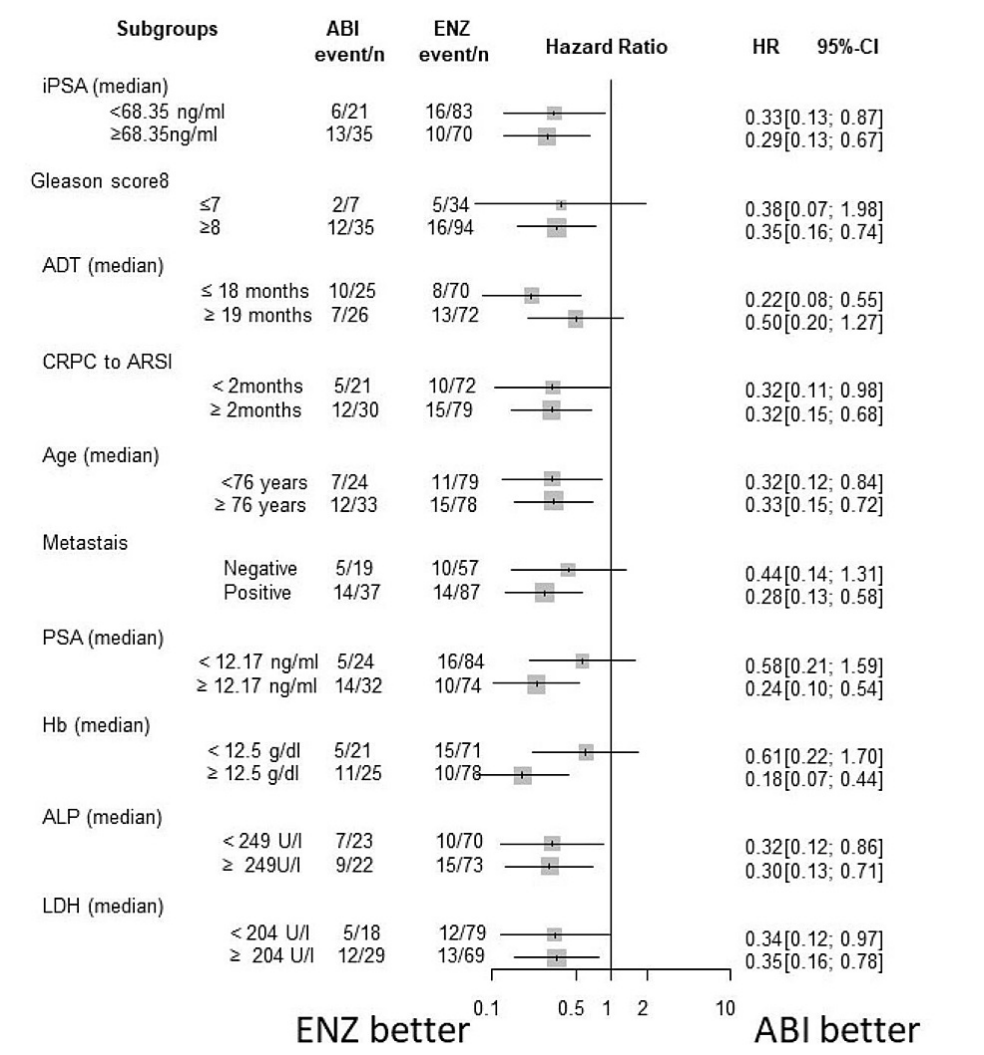
Forrest plot of the subgroup analysis for CSS CSS: cancer-specific survival, iPSA: initial prostate-specific antigen, GS: Gleason score, ADT: androgen deprivation therapy, CRPC: castration-resistant prostate cancer, ARSI: androgen receptor signaling inhibitor, LN: lymph node, PSA: prostate-specific antigen, WBC: white blood cell, Hb: hemoglobin, ALP: alkaline phosphatase, LDH: lactate dehydrogenase, CRP: C-reactive protein

**Figure 18:**
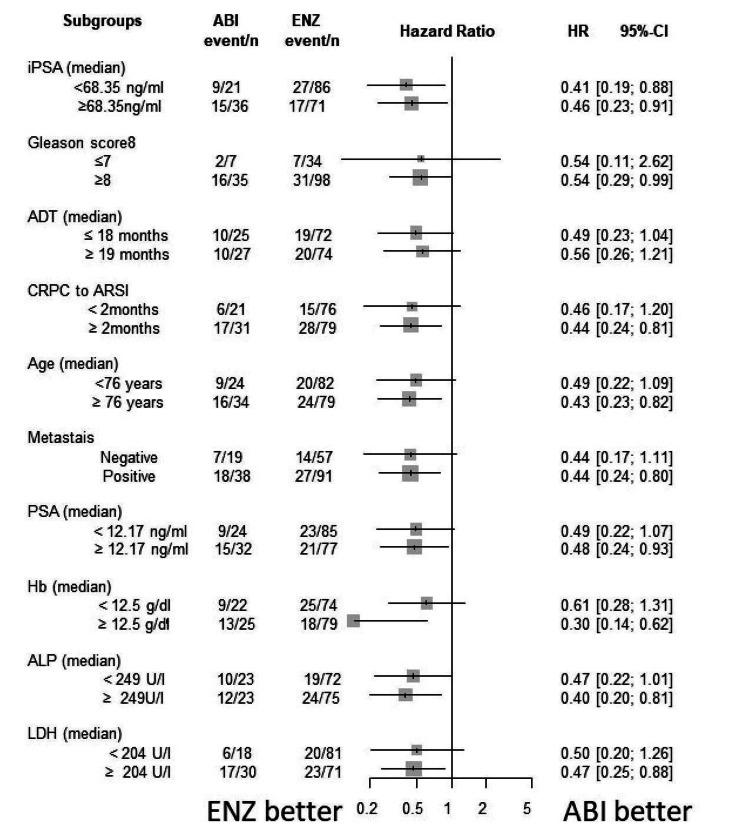
Forrest plot of the subgroup analysis for OS OS: overall survival, iPSA: initial prostate-specific antigen, GS: Gleason score, ADT: androgen deprivation therapy, CRPC: castration-resistant prostate cancer, ARSI: androgen receptor signaling inhibitor, LN: lymph node, PSA: prostate-specific antigen, WBC: white blood cell, Hb: hemoglobin, ALP: alkaline phosphatase, LDH: lactate dehydrogenase, CRP: C-reactive protein

The Forrest plot of HR for PSA-PFS, TFF, CSS, and OS are shown in Figures 15-18. HR of each subgroup is shifted to enzalutamide better in all survival outcomes.

## Discussion

Some reports showed that induction dose of ARSI correlates with oncological outcome [13-15]. Tsuzuki et al. reported that a reduced induction dose of enzalutamide correlated with lower PSA response significantly [13]. PSA response was -87.4% (standard dose) and -66.3% (reduced dose) (p=0.02). Median PFS was 12.1 months (standard dose) and 7.2 months (reduced dose) (p=0.038). Yokomizo et al. reported that a reduced induction dose of enzalutamide correlated with a shorter time for PSA progression in the nmCRPC cohort [14]. Yamada et al. reported that dose reduction was a significant prognostic factor for PFS in multivariate analysis [15]. Median PFS was 12.1 months (standard dose) and 7.2 months (reduced dose) (p=0.038). Recently, we reported the difference in the efficacy between ARSI (abiraterone acetate and enzalutamide) against chemo-naïve CRPC in the real-world setting and concluded that introducing enzalutamide first correlates with better survival outcomes [17]. Although induction dose correlates with the oncological outcome mentioned above, our cohort included both full (standard) dose and reduced dose induction cases. Therefore, we reanalyzed full-dose induction subgroups from our overall cohort to compare the true difference in the efficacy of ARSI against chemo-naïve CRPC in this study. We present the results as follows: (1) PSA decline correlated with survival outcome (PSA-PFS, TFF, CSS, and OS). (2) PSA responses were better in the enzalutamide group. (3) Therefore, survival outcomes (PSA-PFS, TFF, CSS, and OS) were better in the enzalutamide group. The advantages of enzalutamide were also proved in multivariate analysis, IPTW method, and subgroup analysis in this study. PSA response correlates with survival outcomes [17-19], and better PSA response in enzalutamide was proved in a randomized prospective trial [20]. In terms of comparison of survival outcomes between ARSI, although a phase 2 randomized prospective ARSI cross-over trial showed the same OS [20], some meta-analysis studies and two retrospective large cohort studies showed a survival benefit with enzalutamide induction first [21-25]. Although these results were almost the same as our overall cohort [12], the true difference in the efficacy was shown in this full-dose induction analysis. In this study, we focused on the efficacy of ARSI against chemo-naïve CRPC. However, ARSI is introduced in upfront settings (against metastatic hormone-sensitive prostate cancer: mHSPC) nowadays after survival benefits were proved in randomized controlled trials [26-30]. Although the induction timing of ARSI is different between the conventional setting and the upfront setting, the biological feature of prostate cancer would be the same as the failure of first-line ARSI. Therefore, our results could be referred to when considering using ARSI as an upfront setting against mHSPC. Because there is no published prospective trial comparing the survival benefit between ARSI against mHSPC, our results would be informative in selecting the treatment agent. Biologically, the time to CRPC in an upfront setting might be the same as the time to progression after the introduction of the first-line ARSI in a conventional setting. In our study, enzalutamide induction correlated better with PSA-PFS and TFF. Therefore, enzalutamide induction in the upfront setting would correlate with better survival outcomes. Although we could not mention apalutamide because it is not approved against mCRPC, an anti-androgen agent might have more benefit than an androgen synthesis inhibitor against mHSPC.

This study has some limitations. They are the same as our overall cohort study [12]. First, this was a retrospective analysis, the follow-up regimen was not standardized, and the cohort size was relatively small. Moreover, the number of cases in the abiraterone acetate and enzalutamide groups was unbalanced. This was because enzalutamide was approved earlier than abiraterone acetate in Japan.

## Conclusions

The results of this study indicate that the PSA response, PSA-PFS, TFF, CSS, and OS were better with full-dose enzalutamide induction first than with full-dose abiraterone acetate induction. Direct inhibition of AR signaling by enzalutamide was associated with better PSA decline and longer survival outcomes (PSA-PFS, TFF, CSS, and OS).
